# Efficacy of pharmacological interventions and therapeutic exercises for rheumatoid arthritis: a systematic review and meta-analysis

**DOI:** 10.3389/fphar.2025.1686128

**Published:** 2025-11-20

**Authors:** Timothy Adeyemi, Vitalis Ikenna Chukwuike, Motunrayo Adebukunola Oladimeji, Ufuoma Shalom Ahwinahwi, Victor Ayodeji Ayeni, Aisha Adam Abdullahi, Abdulmuminu Isah, Love Bukola Ayamolowo, Oluchukwu Perpetual Okeke, Olunike Abodunrin, Folahanmi Akinsolu, Olajide Odunayo Sobande

**Affiliations:** 1 Department of Physiotherapy, Bowen University, Iwo, Nigeria; 2 Department of Physiotherapy, Bowen University Teaching Hospital, Ogbomosho, Oyo, Nigeria; 3 Department of Industrial and Medicinal Chemistry, David Umahi Federal University of Health Sciences, Uburu, Ebonyi, Nigeria; 4 Department of Anaesthesia, Lagos University Teaching Hospital, Idi-Araba Lagos, Nigeria; 5 Department of Clinical Pharmacy and Pharmacy Administration, Delta State University, Abraka, Nigeria; 6 Department of Paediatrics, Babcock University, Ilishan-Remo, Nigeria; 7 Department of Epidemiology and Population Health, Kano Independent Research Centre Trust, Kano, Nigeria; 8 Department of Clinical Pharmacy and Pharmacy Management, University of Nigeria, Nsukka, Nigeria; 9 Department of Nursing Science, Obafemi Awolowo University, Ife, Nigeria; 10 Nigerian Institute of Medical Research Foundation, Yaba, Lagos, Nigeria; 11 Department of Epidemiology and Biostatistics, Nanjing Medical University, Nanjing, China; 12 Clinical Sciences Department, Lead City University, Ibadan, Nigeria; 13 Nigerian Institute of Medical Research, Yaba, Lagos, Nigeria

**Keywords:** rheumatoid arthritis, pharmacological intervention, therapeutic exercise, adult, efficacy, SDG 3, chronic inflammatory arthritis, auto-immune disease

## Abstract

**Background:**

Rheumatoid arthritis (RA) is a chronic inflammatory autoimmune disease characterized by persistent synovitis, systemic inflammation, and progressive joint damage. Medication and physical exercise are the most common interventions documented for the treatment of RA. The current systematic review examined the efficacy of pharmacological and physical exercise interventions for RA.

**Methods:**

A systematic literature search utilized databases (PubMed, Web of Science, Google Scholar, Scopus, and CINAHL) to identify relevant randomized controlled trials (RCTs). The search strategy included keywords related to rheumatoid arthritis, pharmacological interventions, exercise therapy, and clinical outcomes. The review was registered with PROSPERO (CRD42024587378). Two independent reviewers screened the identified articles, and relevant data were extracted for analysis. The quality of evidence for the outcomes of interest in this systematic review and meta-analysis was assessed using the Grading of Recommendations, Assessment, Development, and Evaluation (GRADE) framework. Meta-analysis was carried out using a random-effects model. For the meta-analysis, we assessed the heterogeneity using the I^2^ statistic.

**Results:**

Eighteen RCTs with a total sample size of 7,062 were included. Of these, eleven studies (61.11%) investigated the efficacy of different pharmacological interventions, whereas seven (38.89%) assessed the effects of exercise interventions. Different exercise programs showed improved functionality, reduced pain, and improved patient quality of life (QoL), while short- and medium-term sensorimotor trainings were reported. Pharmacological interventions also reported varying levels of reduction in disease activity and improved functionality. Pooled effects of four studies included in the meta-analysis revealed the different categories of incremental clinical outcomes (1.89, 2.46, and 2.63) after 24 months based on the American College of Rheumatology (ACR) criteria—ACR 20, ACR 50, and ACR 70, respectively. Significant effect sizes (0.09 and −0.08) after 24 weeks were equally found in three studies, indicating the reduction in disease activity and an improvement in functionality, respectively. Despite robust efficacy data, a major limitation across the literature is the heterogeneity of reported ACR outcomes, attributable to pharmacological variation (immunogenicity and disease stage) rather than purely statistical bias.

**Conclusion:**

Pharmacological and physical exercise interventions benefit many patients with RA, particularly regarding clinical outcomes such as reduced disease activity and improved functional ability; and both interventions are potentially synergistic in RA. However, more RCTs are required, especially in Sub-Saharan Africa (SSA), to buttress the evidence base for these interventions. Crucially, the review notes the need for greater pharmacological discussion on safety and tolerability in future studies.

**Systematic Review Registration:**

https://www.crd.york.ac.uk/PROSPERO/view/CRD42024587378.

## Introduction

Rheumatoid Arthritis (RA) is an autoimmune disease with a prevalence of 0.5–1.0% in adults, causing pain, swelling, and/or deformations of hands, wrists, and feet joints, leading to a reduction in functional ability and quality of life (QoL) (Kvien, 2004; Matcham et al., 2014). The most important symptoms include debilitating pain, joint tenderness, swelling, morning stiffness, and functional limitations leading to disability. The global burden of RA has increased over the past decades and is postulated to continue increasing in the coming years, necessitating more attention to optimal treatment worldwide (Cai et al., 2023; Strand and Khanna, 2010). The global age-standardized prevalence rate of rheumatoid arthritis increased from 207.46 cases per 100,000 population (95% uncertainty interval: 189.99–226.95) in 1990 to 224.25 cases per 100,000 population (95% UI: 204.94–245.99) in 2019, indicating an increasing trend over nearly three decades (Cai et al., 2023). Chronic pain, which is a well-known debilitating feature of RA, characterized by its persistence and intensity, can significantly impair an individual’s quality of life, resulting in emotional distress, functional limitations, and reduced social participation (Strand and Khanna, 2010; Guo et al., 2018; Yildizeli Topcu, 2018).

Rheumatoid arthritis presents a challenging medical issue due to its recurrent and systemic inflammatory course (Cai et al., 2023). When disease activity is not rapidly and effectively controlled, this could lead directly to progressive joint damage and chronic pain; this sustained physical impairment results in a high disability rate and significant limitations in the patient’s capacity to work. The resultant loss of labor productivity is a major component of the overall high socioeconomic burden (Cai et al., 2023; Strand and Khanna, 2010; Yildizeli Topcu, 2018). The management of RA typically involves pharmacological interventions, such as disease-modifying antirheumatic drugs (DMARDs), non-steroidal anti-inflammatory drugs (NSAIDs), corticosteroids, and biologics, which aim to reduce inflammation and halt disease progression (Guo et al., 2018; Kumar and Banik, 2013). Pharmacological interventions, according to the European Union Directive 2001/83/EC, can be defined as follows: “any substance or combination of substances which may be used in or administered to human beings either to restore, correct or modify physiological functions by exerting a pharmacological, immunological or metabolic action, or to make a medical diagnosis” (Dunn et al., 2011). In addition, structured physical exercise for therapeutic purposes has emerged as a complementary approach to managing RA. Exercise programs, including aerobic and resistance training, have improved physical function, reduced pain, and enhanced quality of life in individuals with RA (Geneen et al., 2017). Therapeutic exercise offers a non-pharmacological approach that addresses the underlying musculoskeletal impairments associated with RA, such as joint stiffness, muscle weakness, and impaired range of motion (Yamada et al., 2017), improving pain perception, enhancing functional capacity, and promoting psychological wellbeing in individuals with RA (Yildizeli Topcu, 2018).

Despite the widespread use of pharmacological treatments (Nam et al., 2017; Donahue et al., 2008; Kerschbaumer et al., 2020; Hazlewood et al., 2020) to manage inflammation and alleviate symptoms, many patients experience functional limitations. Therapeutic exercises are increasingly recognized as complementary to enhancing mobility, improving physical function, and potentially mitigating joint damage (Sobue et al., 2022; Baillet et al., 2012; Cairns and McVeigh, 2009; Ye et al., 2022; Bergstra et al., 2014; Boniface et al., 2020). However, the evidence regarding their efficacy and how they compare to pharmacological interventions remains inconsistent and poorly synthesized.

This systematic review aimed to evaluate the efficacy of pharmacological and therapeutic exercises, as stand-alone or combination interventions, in managing pain, improving functionality, and enhancing QoL in adults with RA. The findings are particularly relevant to low- and middle-income countries (LMICs), where optimized care strategies could alleviate the financial burden of managing this complex and prevalent autoimmune disease, where healthcare funding is done out-of-pocket while enhancing clinical decision-making, improving management approaches, and guiding future research to advance precision and effectiveness in RA care.

## Methods

The review was registered with PROSPERO (CRD42024587378), and the study was performed and reported using the guidelines for Preferred Reporting Items for Systematic Reviews and Meta-analysis (PRISMA). The protocol was guided by previous reviews and meta-analyses on the pharmacological and therapeutic exercise management of RA (Hazlewood et al., 2020; Ye et al., 2022).

### Literature search

PubMed, Web of Science, Google Scholar, Scopus, and CINAHL for articles published between 1 January 2004 and 31 August 2024 were systematically searched (search strategy details are in Supplementary File S1). The search terms included keywords relating to “Rheumatoid Arthritis,” “Pharmacological Intervention,” “Therapeutic Exercise,” and “Adult.” The study search was global in scope and not restricted by geographical coverage. However, it was limited to studies published in English within the last 21 years to ensure the inclusion of recent data on the efficacy of clinical interventions for rheumatoid arthritis (Supplementary Datasheet 1).

### Selection criteria

Using a set of inclusion and exclusion criteria, the final analysis of randomized controlled trials (RCTs) published in peer-reviewed journals was included. RCTs were eligible to be included if they were conducted on RA, had a protocol for the clinical trial, and had a compatible intervention (drug or exercise) and comparator, according to the Population, Intervention, Comparators, Outcomes, Time, Studies (PICOTS) framework (Feldner and Dutka, 2024). Table 1 details the PICOTS framework used for this study. Studies were excluded if they included secondary data in the analysis of findings from their clinical trials or if they were switch trials, strategy studies, study protocols, double trials, or contained insufficient participant data. Other excluded studies were those with incompatible interventions and/or outcomes listed as comparators, those that recruited participants under 18 years, those that were abstracts only, or those that were incomplete. In addition, studies were excluded if full texts were not available online or if they were published before January 2004. The articles were screened initially by titles and abstracts, followed by full-text screening for studies available in full texts.

**TABLE 1:** Eligibility criteria using the Population, Intervention, Comparisons, Outcomes, Time, and Studies (PICOTS) framework.

Population	All studies in the English language evaluated the efficacy of pharmacological and/or therapeutic exercise interventions on adults with rheumatoid arthritis aged 18 years and above
Intervention	Pharmacological intervention or therapeutic exercise
Comparators	Usual care, alternative medications, other drug dosage(s), and other exercise types
Outcomes	1. Pain 2. Quality of life 3. Health-related quality of life 4. Disability 5. Physical function 6. Resolution of stiffness and/or inflammation
Time	01/01/2004 to 08/31/2024
Studies	Randomized controlled trials (RCTs)

### Data extraction

The selection of studies was done in phases using the inclusion and exclusion criteria. Two authors (USA and VIC) independently screened the titles and abstracts of the articles. Inter-rater reliability was performed between the two authors, with initial kappa (r) values of 0.49 prior and 1.00 post-conflict resolution, to ensure compatibility in the authors’ screening (Supplementary Table 3). At this screening phase, resolution of conflict by a third author (MAO) was ensured, and agreement between the two authors was achieved. The full articles of those deemed eligible were thereafter retrieved and independently screened by four authors (MAO, USA, VIC, and TA), with the first listed three authors doing the full-text screening (two authors per paper) and the fourth (TA) arbitrating and facilitating resolution, where there was any conflict.

The data from the included studies were extracted using a pretested tool developed by the authors. Information retrieved included the first author’s surname, the year of publication, study setting, disease severity, disease duration, blinding status, study type, sample size enrolled, sample size of the intervention group(s), the sample size of the control group(s), the sample size that completed the study in intervention and control group(s), male and female individuals in intervention and control group(s), mean age (where applicable) for intervention and control group(s), median age (where applicable) for intervention and control group(s), report on missing data; intervention type, specific intervention, duration of intervention, and comparator(s); baseline data/outcome(s), the primary outcome(s); secondary outcome(s), data analysis tools; the participants’ use of background drugs; assessment tools; number of assessments; times of assessments; and follow-up (Supplementary Table 1). The extracted data were thereafter randomly selected and cross-checked following the listing on the sheet (Supplementary Datasheet 1).

### Quality assessment

The quality of the papers was assessed using the Joanna Briggs Institute Critical Appraisal Checklist for randomized controlled trials (Barker et al., 2023). The checklist assesses the methodological quality of RCTs based on 13 questions (Supplementary Table 2). Possible responses were “yes,” “no,” “unclear,” or “not applicable.” A maximum score of 1 was assigned to each question, with a potential minimum score of 0 and a maximum of 9. However, we decided, *ab initio*, not to exclude any study based on the quality rating.

### Statistical analysis

Meta-analysis was carried out using a random-effects model, known for its usefulness in presenting the extent of variation between studies (DerSimonian and Laird, 1986). For each meta-analysis, we assessed heterogeneity using the I^2^ statistic and publication bias using funnel plots (Higgins et al., 2003; Egger et al., 1997).

Meta-analysis was conducted when three or more included studies reported the same outcome/intervention. Risk ratios (RR) and 95% confidence intervals (CIs) were pooled using a random-effects model, specifically applying the DerSimonian and Laird estimator (DerSimonian and Laird, 1986). Furthermore, the standardized mean difference (SMD) was calculated using a random-effects model for studies that reported mean. Cochran’s test was used to assess any discrepancy or heterogeneity. Heterogeneity was assessed using the I^2^ statistic, a widely used measure in meta-analysis to quantify the degree of heterogeneity and indicate the inconsistency or variability in results across the studies included in the meta-analysis (Higgins et al., 2003; Higgins and Thompson, 2002). The I^2^ value ranges from 0% to 100%, with higher values indicating greater heterogeneity (Points et al., 2019; Borenstein et al., 2009). A p-value of <0.01 for the Q test and an I^2^ value greater than 50% indicated statistically significant heterogeneity. Sensitivity analysis was conducted using the trim-and-fill method (Duval and Tweedie, 2000). The data were analyzed using R Studio version 4.2.2.

### GRADE assessment of evidence

The quality of evidence for the outcomes of interest in this systematic review and meta-analysis was assessed using the Grading of Recommendations, Assessment, Development, and Evaluation (GRADE) framework to evaluate the certainty of evidence systematically across five key domains, namely, risk of bias, inconsistency, indirectness, imprecision, and publication bias (Guyatt et al., 2011; Guyatt et al., 2013). We adhered to standardized GRADE protocols in rating the evidence for all the study outcomes.

### GRADE assessment process

**FIGURE 1:**
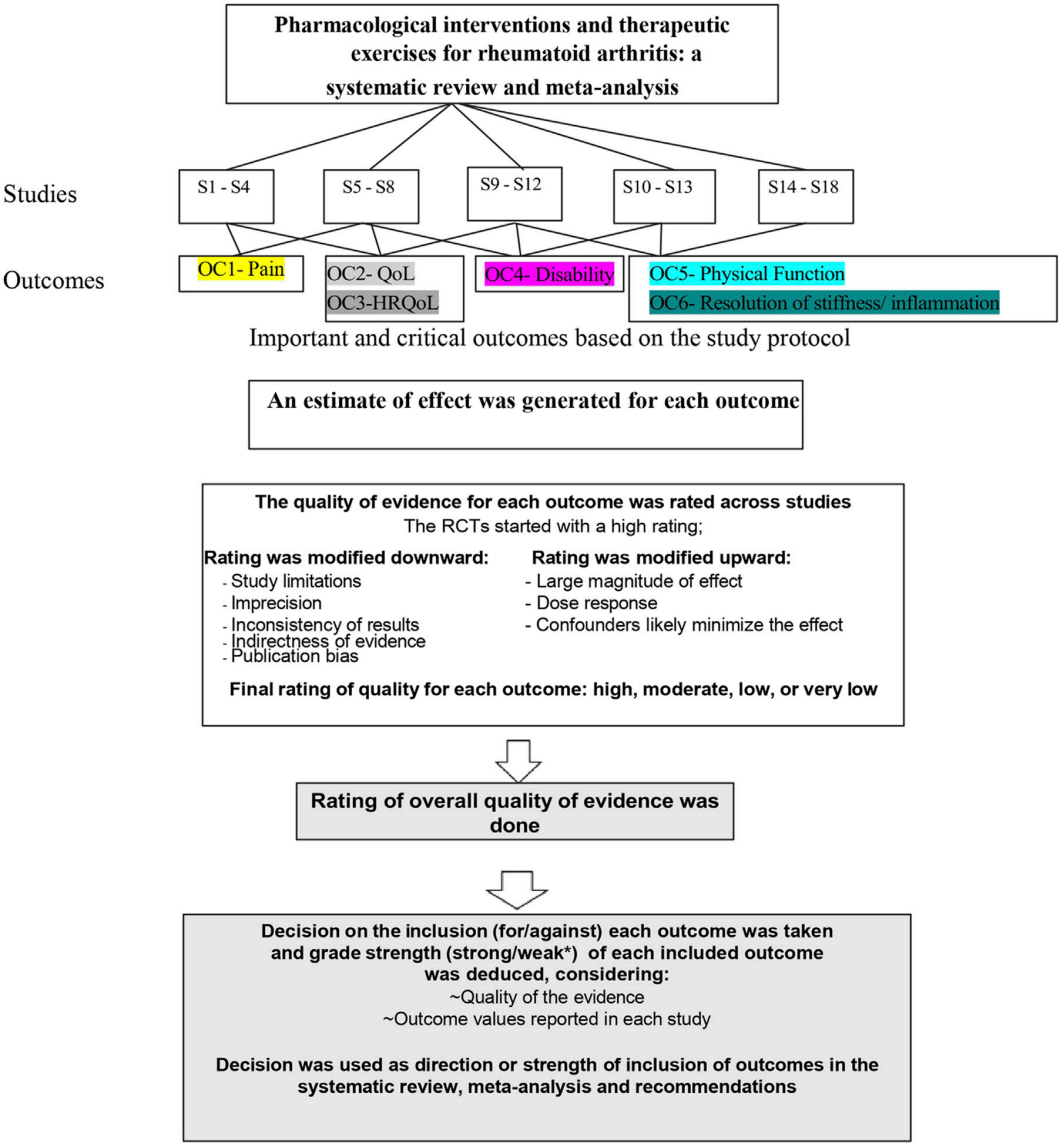
A schematic diagram of GRADE’s process used for the inclusion of study outcomes in the systematic review, meta-analysis, and recommendations. Abbreviations: RCT, randomized controlled trials; QoL, quality of life; HRQoL, health-related quality of life, S; study.

Two independent reviewers assessed each included study against the GRADE domains, with resolution of any disagreement done by a third reviewer who served as an arbitrator. The quality of evidence for each outcome in all included RCTs was rated down if they were found wanting in each of the aforementioned five domains and rated up if the studies recorded a large magnitude of effect, large dose–response gradient for the outcome, and/or notable consideration of the effect of all plausible residual confounders denoting an increase in the estimated effect (Figure 1).

The GRADE ratings of quality of evidence for our study were determined as follows:

1. High: No serious flaws in study quality.
2. Moderate: Serious flaws in design and execution.
3. Low: Very serious flaws in design or execution.
4. Very low: Very serious flaws and at least one other serious threat to validity

## Results

### Study characteristics

**FIGURE 2:**
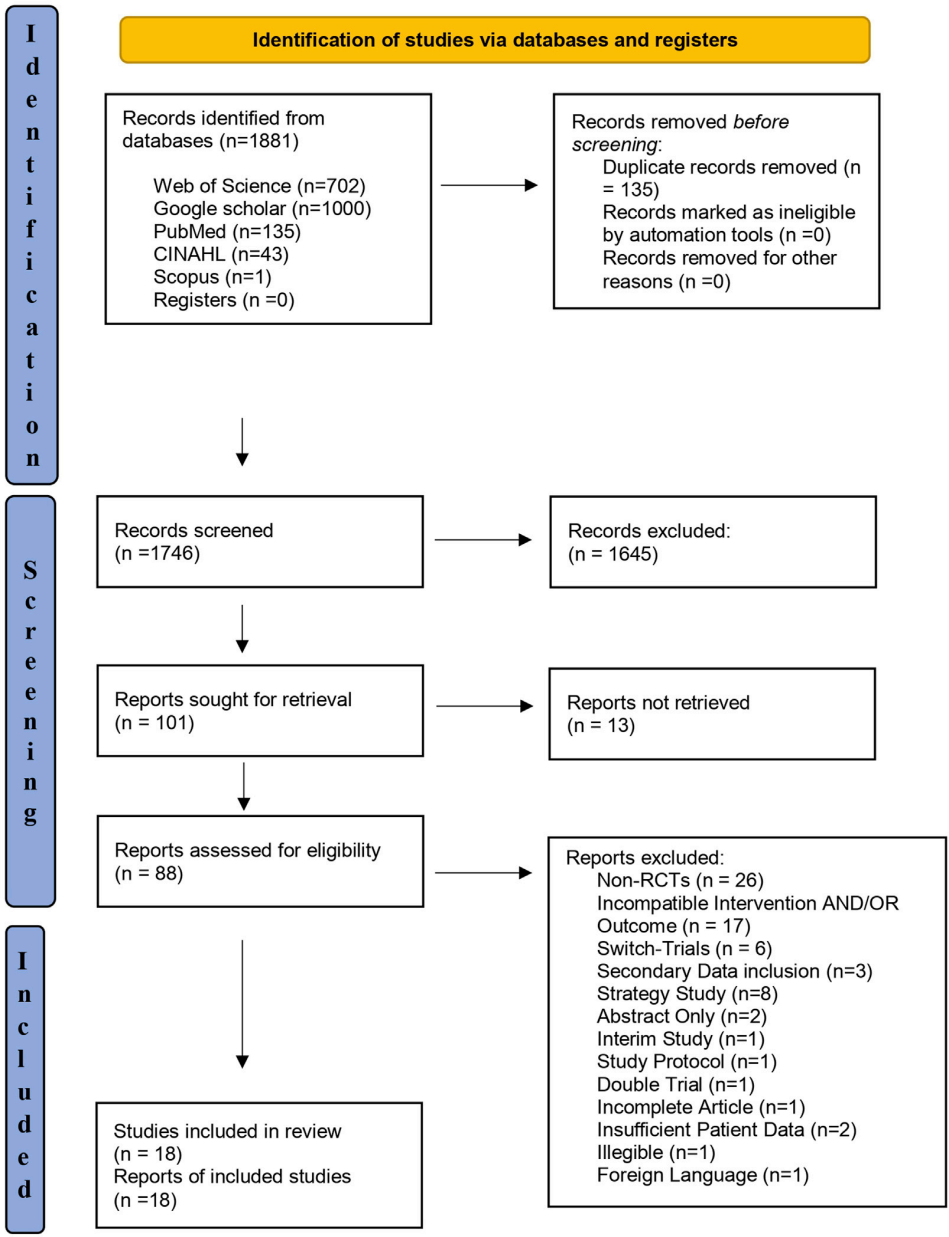
PRISMA flow diagram for pharmacological interventions and therapeutic exercises for rheumatoid arthritis.

Figure 2 shows the PRISMA flow diagram for study selection. One thousand eight hundred and eighty-one studies were retrieved through electronic searches of the four databases. After removing duplicates, 1,746 articles remained, and their titles and abstracts were screened. Thereafter, 1,645 articles were found irrelevant and excluded. The full-text records for the remaining 101 studies were sought and assessed for detailed evaluation, after which 70 articles were excluded based on reasons illustrated in Figure 2. Finally, 18 studies met the selection criteria, and the included studies’ characteristics are summarized in Table 2.

**TABLE 2:** Summary characteristics of studies included in the review, 2004–2024.

S/N	Author name	Study year	Title	Sample size	Study country	Mean age (SD)			Risk rating
						Control	Intervention 1	Intervention 2	
1	Teuwen et al. (2024)	2024	Effectiveness of longstanding exercise therapy compared with usual care for people with rheumatoid arthritis and severe functional limitations: a randomized controlled trial	215	Netherlands	58.1 (13.6)	59.4 (12.1)	--	Low
2	Rodríguez Sánchez-Laulhé et al. (2022)	2022	An Exercise and Educational and Self-management Program Delivered With a Smartphone App (CareHand) in Adults With Rheumatoid Arthritis of the Hands: Randomized Controlled Trial	36 (69 Hands)	Spain	61.86 (10.76)	57.64 (7.25)	--	Low
3	Da Silva et al. (2013)	2013	Effectiveness of sensorimotor training in patients with rheumatoid arthritis: a randomized controlled trial	102	Brazil (Sao Paulo)	58.37 (8.11)	57.90 (8.50)	--	Low
4	Klareskog et al. (2004)	2004	The therapeutic effect of the combination of etanercept and methotrexate compared with each treatment alone in patients with rheumatoid arthritis: double-blind randomized controlled trial	682	International Multicenter	53·0 (12·8)	52·5 (12·4	53.2 (13.8)	Low
5	Häkkinen et al. (2004)	2004	Sustained maintenance of exercise-induced muscle strength gains and normal bone mineral density in patients with early rheumatoid arthritis: a 5-year follow-up up	70	Finland	49 (11)	49 (10)	--	Moderate
6	Lamb et al. (2015)	2015	Exercises to improve function of the rheumatoid hand (SARAH): a randomized controlled trial	490	United Kingdom	63·5 (11)	61·3 (12	--	Low
7	Van Den Berg et al. (2006)	2006	Using Internet technology to deliver a home-based physical activity intervention for patients with rheumatoid arthritis: A randomized controlled trial	160	Netherlands-based multicenter	49.8 (13.9)	−49.5 (12.9)	--	Low
8	Kremer et al. (2006)	2006	Effects of batacept in patients with methotrexate-resistant active rheumatoid arthritis: a randomized trial	652	Multicenter (26 countries)	50.4 (12,4)	51.5 (12.9)	--	Low
9	Emery et al. (2008)	2008	Comparison of methotrexate monotherapy with a combination of methotrexate and etanercept in active, early, moderate to severe rheumatoid arthritis (COMET): a randomized, double-blind, parallel treatment trial	528	Europe, Asia, Latin America, and Australia	52.3 (0.8)	50.5 (0.9)	--	Low
10	Genovese et al. (2008)	2008	Interleukin-6 receptor inhibition with Tocilizumab reduces disease activity in rheumatoid arthritis with inadequate response to disease-modifying anti-rheumatic drugs: the Tocilizumab in combination with traditional disease-modifying antirheumatic drug therapy study	1,216	Multicenter	54 (13)	53 (13)	--	Low
11	Smolen et al. (2008)	2008	Effect of interleukin-6 receptor inhibition with Tocilizumab in patients with rheumatoid arthritis (OPTION study): a double-blind, placebo-controlled, randomized trial	620	--	50.6 (12.1)	50.8 (11.8)	51.4 (12.8)	Low
12	Fleischmann et al. (2009)	2009	Efficacy and safety of Certolizumab pegol monotherapy every 4 weeks in patients with rheumatoid arthritis failing previous disease-modifying antirheumatic therapy: the FAST4WARD study	220	Multicenter	54.9 (11.6)	52.7 (12.7)	--	Low
13	Gabay et al. (2013)	2013	Tocilizumab monotherapy versus adalimumab monotherapy for the treatment of rheumatoid arthritis (ADACTA): a randomized, double-blind, controlled phase 4 trial	326	Multicenter (76 countries)	54.4 (13.0)	53.3 (12.4)	--	Low
14	van Vollenhoven et al. (2009)	2009	Addition of Infliximab compared with the addition of sulfasalazine and hydroxychloroquine to methotrexate in patients with early rheumatoid arthritis (Swefot trial): 1-year results of a randomized trial	258	Sweden	52.9 (13.9)	51.1 (13.3)	--	Moderate
15	Smolen et al. (2009)	2009	Efficacy and safety of certolizumab pegol plus methotrexate in active rheumatoid arthritis: the RAPID 2 study. A randomized controlled trial	619	International Multicenter Study	51.5 (11.8)	51.9 (11.8)	52.2 (11.1)	Low
16	Nishimoto et al. (2004)	2004	Treatment of Rheumatoid Arthritis With Humanized Anti–Interleukin-6 Receptor Antibody: A Multicenter, Double-Blind, Placebo-Controlled Trial	162	Japan	53.0 (31–70)	56.0 (25–74)	53.5 (3–74)	Low
17	Lourenzi et al. (2017)	2017	Effectiveness of an overall progressive resistance strength program for improving the functional capacity of patients with rheumatoid arthritis: a randomized controlled trial	60	Brazil	50.88 (8.57)	52.63 (7.10)	--	Low
18	Schiff et al. (2014)	2014	Head-to-head comparison of subcutaneous abatacept versus adalimumab for rheumatoid arthritis: 2-year efficacy and safety findings from AMPLE trial	646	International Multicenter Study	51.0 (12.8)	51.4 (12.6)	--	Low

SD - Standard Deviation

### Characteristics of included studies

Eighteen studies (Table 2) were included, with a total sample size of 7,062 patients with RA. All (100%) of the studies were facility-based RCTs (Cairns and McVeigh, 2009; Ye et al., 2022; Bergstra et al., 2014; Boniface et al., 2020; Feldner and Dutka, 2024; Barker et al., 2023; DerSimonian and Laird, 1986; Higgins et al., 2003; Egger et al., 1997; Higgins and Thompson, 2002; Points et al., 2019; Borenstein et al., 2009; Duval and Tweedie, 2000; Guyatt et al., 2011; Guyatt et al., 2013; Teuwen et al., 2024; Rodríguez Sánchez-Laulhé et al., 2022; Da Silva et al., 2013). The review showed that more attention has been paid to pharmacological intervention than exercise therapies. However, most of the recent articles are based on therapeutic exercise. Seven studies (38.89%) focused on therapeutic exercise (Teuwen et al., 2024; Rodríguez Sánchez-Laulhé et al., 2022; Da Silva et al., 2013; Häkkinen et al., 2004; Lamb et al., 2015; Van Den Berg et al., 2006; Lourenzi et al., 2017), while the remaining 11 studies (61.11%) had varying pharmacological interventions as their intervention of interest (Boniface et al., 2020; Higgins et al., 2003; Egger et al., 1997; Higgins and Thompson, 2002; Points et al., 2019; Borenstein et al., 2009; Duval and Tweedie, 2000; Guyatt et al., 2011; Guyatt et al., 2013; Teuwen et al., 2024; Da Silva et al., 2013).

The intervention groups’ age range was 49.0–61.3 years, while the controls were 49.0–63.5 years. It is worth noting that all the intervention and control groups were on methotrexate (MTX) as a background medication. A geographical survey of the studies shows that eight studies (44.44%) were conducted in single centers in individual countries (Teuwen et al., 2024; Rodríguez Sánchez-Laulhé et al., 2022; Da Silva et al., 2013; Häkkinen et al., 2004; Lamb et al., 2015; van Vollenhoven et al., 2009; Nishimoto et al., 2004; Lourenzi et al., 2017), three were multicenter studies, each conducted in a single country (16.67%) (Van Den Berg et al., 2006; Genovese et al., 2008; Fleischmann et al., 2009), six were international multicenter studies (33.33%) (Klareskog et al., 2004; Kremer et al., 2006; Emery et al., 2008; Gabay et al., 2013; Smolen et al., 2009; Schiff et al., 2014), and one study (5.56%) had its geographical center unstated (Smolen et al., 2008).

### Risk-of-bias assessment of included studies

The included studies were assessed for methodological quality and risk of bias using the Joanna Briggs Institute (JBI) Critical Appraisal Checklist for Randomized Controlled Trials. Studies with a JBI rating of ≤50 were rated as having a high risk of bias; those with a rating of 51–74 as moderate risk; and those with a rating of ≥75 as having a low risk of bias. No study was deemed to have a high risk of bias. Sixteen studies had a low risk of bias (88.9%), and two had a moderate risk of bias (11.1%) (S4 File).

### Meta-analysis

A meta-analysis was conducted to assess the overall efficacy of the interventions using a broader sample size than each of the individual studies that investigated clinical outcomes, change in disease activity, and improvement in functional disability.

**FIGURE 3:**
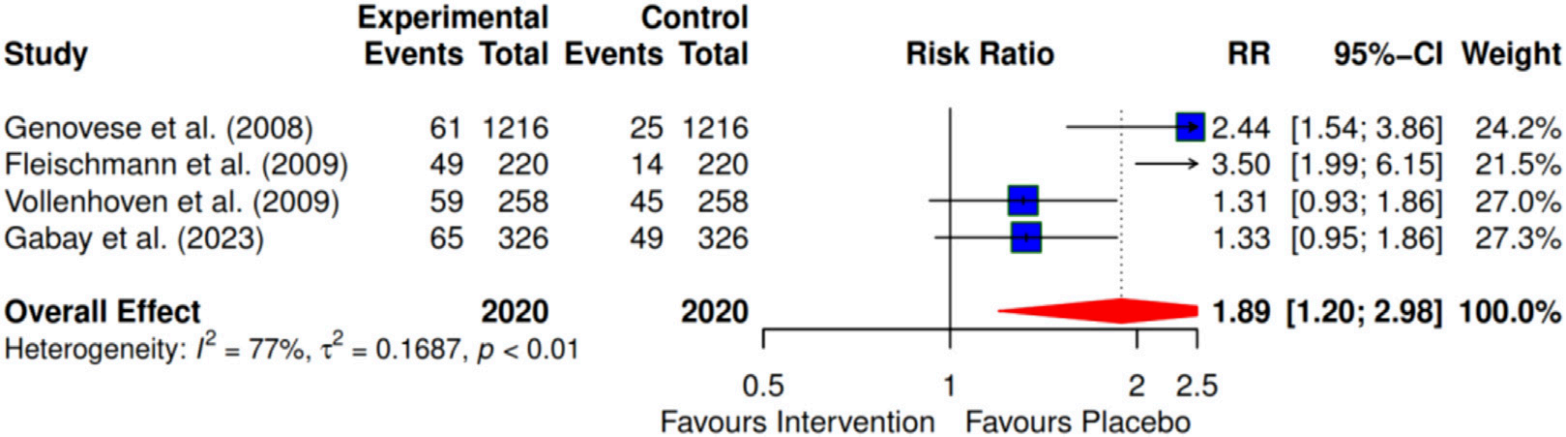
Forest plot showing ACR 20 at 24 months. The overall effect shows that the intervention (DMARDs) significantly favours the treatment group with a pooled Risk Ratio (RR) of 1.89 (95% CI, 1.20–2.98).

**FIGURE 4:**
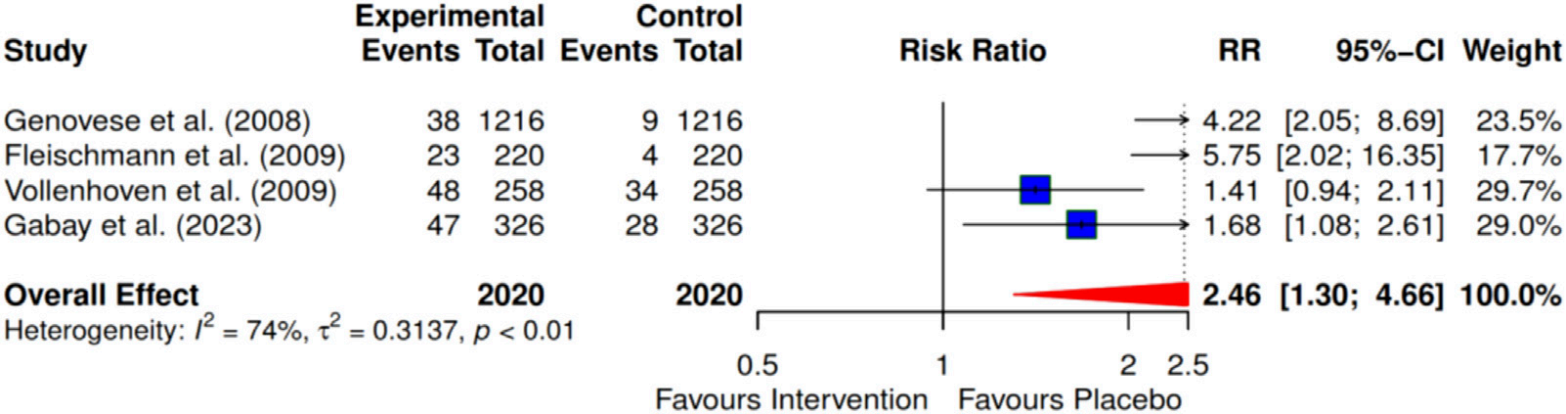
Forest plot showing ACR 50 at 24 months. The overall pooled Risk Ratio is 2.46 (95% CI, 1.30–4.66), demonstrating significant efficacy for the intervention.

**FIGURE 5:**
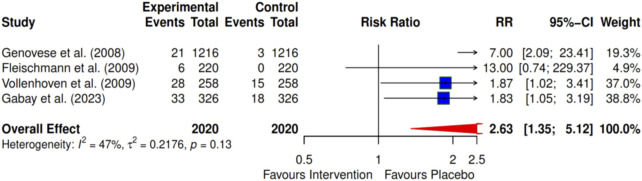
Forest plot showing ACR 70 at 24 months. At near-remission (ACR 70), the pooled Risk Ratio is 2.63 (95% CI, 1.35–5.12), showing a significant benefit for the pharmacological intervention.

Four of the included studies investigated the effects of intervention on the American College of Rheumatology (ACR) criteria ACRs 20, 50, and 70 at 24 months. The meta-analysis indicates a significant improvement with the ACR 20 intervention, showing an RR of 1.86 (95% CI: 1.20–2.98; I^2^ = 77%) (Figure 3). For ACR 50, the RR was 2.46 (95% CI: 1.30–4.66; I^2^ = 74%) (Figure 4), and for ACR 70, the RR was 2.63 (95% CI: 1.35–5.12; I^2^ = 47%) (Figure 5). Egger’s test revealed significant publication bias for ACRs 20 and 50, with p-values of 0.0072 and 0.035, respectively. In contrast, ACR 70 showed no evidence of publication bias, with a p-value of 0.1045 (Figures 3–5).

Using the trim-and-fill method, we found that the effects for ACR 20 and ACR 50 remained inconsistent after conducting sensitivity analyses. However, ACR 70 demonstrated consistent results after these analyses.

**FIGURE 6:**
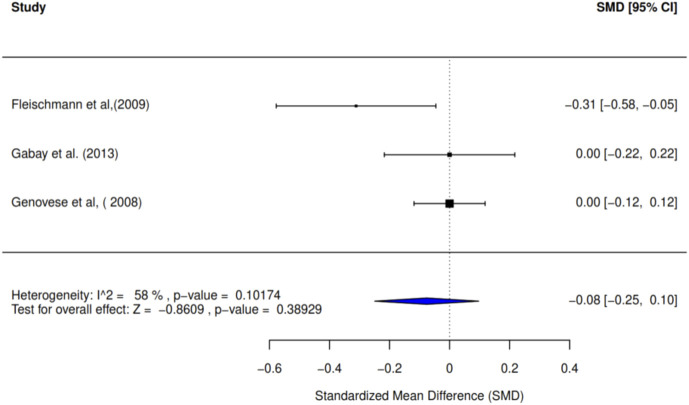
Forest plot showing SMD of HAQ-DI at 24 weeks. The overall effect shows a non-significant, small reduction in disability (SMD –0.08) compared to the control group.

Three studies were assessed by intervention and control groups (IG and CG) to evaluate the effect of the intervention on the Health Assessment Questionnaire Disability Index (HAQ-DI) at 24 weeks. As illustrated in Figure 6, the standardized mean difference is −0.08 (95% CI: −0.25 to 0.10; I^2^ = 58%). This indicates no significant difference between the intervention and control groups, as reflected by a p-value of 0.389 (Figure 6).

**FIGURE 7:**
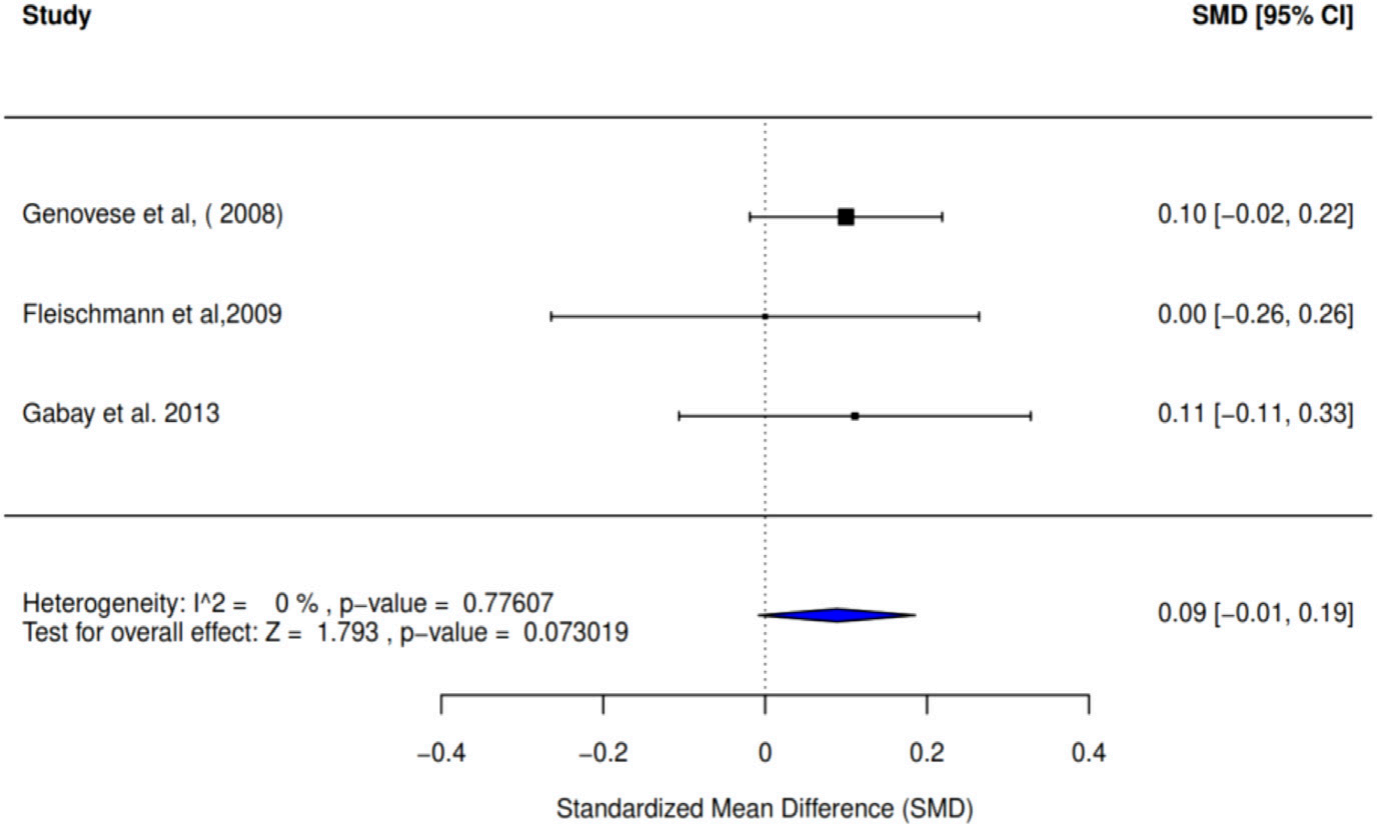
Forest plot showing SMD of DAS at 24 weeks. The overall effect shows a small, non-significant reduction in disease activity (SMD 0.09) compared to the control group.

Furthermore, we conducted a meta-analysis of three studies to evaluate the effect of intervention on the Disease Activity Score (DAS). The pooled results yielded an SMD of 0.09 (95% CI: −0.01 to 0.19; I^2^ = 0%), with no publication bias. This finding suggests no statistically significant difference between the intervention and control groups (p-value = 0.07) (Figure 7). The summary of the effect sizes of the individual articles is presented in Table 3, showing the contribution of each intervention as administered either as monotherapy or in combination.

**TABLE 3:** Summary of meta-analysis findings.

Author/year	Label		Number of participants at post-intervention	Statistical parameter	Outcome	Effect size (95% CI)	P-value	I^2^ (%)
	IG	CG						
Genovese et al. (2008)	Tocilizumab + DMARDs) (IL-6 receptor inhibitor)	Placebo + DMARDs	2,432	RR	ACR 20	2.44 (1.54–3.86)	<0.01	77
					ACR 50	4.22 (2.05–8.69)	<0.01	74
					ACR 70	7.00 (2.09–23.41)	= 0.13	47
Fleischmann et al. (2009)	Certolizumab pegol (TNF inhibitor)	Placebo	440	RR	ACR 20	3.50 (1.99–6.15)	<0.01	77
					ACR 50	5.75 (2.02–16.35)	<0.01	74
					ACR 70	13.00 (0.74–229.37)	= 0.13	47
van Vollenhoven et al. (2009)	Infliximab + MTX) (TNF inhibitor + csDMARD)	Sulfasalazine + hydroxychloroquine + MTX (triple csDMARD)	516	RR	ACR 20	1.31 (0.93–1.86)	<0.01	77
					ACR 50	1.41 (0.94–2.11)	<0.01	74
					ACR 70	1.87 (1.02–3.41)	= 0.13	47
Gabay et al. (2013)	Tocilizumab (IL-6 receptor inhibitor)	Adalimumab (TNF inhibitor)	652	RR	ACR 20	1.33 (0.95–1.86)	<0.01	77
					ACR 50	1.68 (1.08–2.61)	<0.01	74
					ACR 70	1.83 (1.05–3.19)	= 0.13	47
Fleischmann et al. (2009)	Certolizumab pegol (TNF inhibitor)	Placebo	104	SMD	HAQ-DI	−0.31 (0.58–0.05)	= 0.38929	58
Gabay et al. (2013)	Tocilizumab (IL-6 receptor inhibitor)	Adalimumab (TNF inhibitor)	274	SMD	HAQ-DI	0.00 (−0.22-0.22)	= 0.38929	58
Genovese et al. (2008)	Tocilizumab (IL-6 receptor inhibitor)	Placebo	1,121	SMD	HAQ-DI	0.00 (−0.12-0.12)	= 0.38929	58
Genovese et al. (2008)	Tocilizumab (IL-6 receptor inhibitor)	Placebo	1,121	SMD	DAS 28	0.10 (−0.02-0.22)	= 0.073019	0%
Fleischmann et al. (2009)	Certolizumab pegol (TNF inhibitor)	Placebo	104	SMD	DAS 28	0.00 (−0.26-0.26)	= 0.073019	0%
Gabay et al. (2013)	Tocilizumab (IL-6 receptor inhibitor)	Adalimumab (TNF inhibitor)	274	SMD	DAS 28	0.11 (−0.11-0.33)	= 0.073019	0%

DMARD, disease-modifying antirheumatic drug; IG, intervention group, CG, –control group; RR, risk ratio; SMD, standardized mean difference; CI, confidence interval; ACR, American College of Rheumatology; DAS 28, disease-activity score based on 28 joint count; HAQ-DI, Health Assessment Questionnaire–Disability Index; csDMARD, conventional synthetic disease-modifying antirheumatic drug.

### GRADE assessment results—application to study outcomes

The GRADE assessment was applied to all the study outcomes—pain, quality of life, health-related quality of life (HRQoL), disability, physical function, and resolution of stiffness and/or inflammation. The results of this assessment are summarized in Table 4, which presents the final GRADE ratings for each outcome, with justifications for upgrading or downgrading the quality of evidence. This GRADE enhances a clear separation between the quality of evidence and strength of recommendation and provides a transparent process to move from evidence to recommendations concerning the study outcomes. The quality of evidence of the study outcomes was assessed using the GRADE framework, supporting the review and meta-analysis findings. As applicable, the summary of information on potential biases, inconsistencies, indirectness, imprecision, and publication biases is given in Table 4 and reported as follows.

1. Pain: The evidence for joint pain outcomes in the included studies was of moderate quality across all four studies, as examined through 24 months using the American College of Rheumatology Criteria (ACR 20 and ACR 50) due to observed publication bias (coupled with pronounced heterogeneity and large confidence intervals). Although there was some inconsistency in the sensitivity analysis, this was not large enough to lower the rating of the outcomes further as the RCTs were well reported in the individual studies. Moreover, using ACR 70, evidence for joint pain outcomes was high, owing to the elimination of publication bias, observed consistency in the sensitivity analysis, and below-average heterogeneity. The findings apply to the target population of individuals with RA as the studies measured pain in a large number of patients across varying multi-center settings.
2. Physical function: The evidence for physical function in the included studies was of moderate quality in all four studies, as over 24 months according to ACR 20 and ACR 50, largely owing to publication bias, high heterogeneity (77% and 74%, respectively), and large confidence intervals. Furthermore, evidence for physical function was high, as observed with the ACR 70 over the 24 months, with reduced heterogeneity (I^2^: 47%). These outcome findings are largely generalized across studies from similar populations in different countries.
3. QoL and HRQoL: Similar to the outcomes of pain and physical function as measured by the ACR, evidence for the Qol and HRQoL was of moderate quality, using ACR 20 and ACR 50, and high quality using ACR 70 with a strong basis of representativeness of the study population. The absence of publication bias with ACR 70 suggests that the variable measurement at that criterion level had a balanced representation of studies.
4. Disability level: As evidenced by the HAQ-DI, the rating of the disability outcome included in the studies was high, with no detection of publication bias across the three studies, with measurement spanning a 24-week duration. In furtherance of the study outcome strength, the significant mean difference was low (−0.08), with approximately average heterogeneity (I^2^: 58%) across the pooled studies. The balance of study types across the various multicenter studies from different countries contributed largely to the strength of the evidence from these studies. The RCT sources of the disability study outcome were notably well-executed clinical trials with well-reported study details, increasing their reliability and reproducibility.
5. Stiffness: The evidence rating for the study outcome of stiffness and markers of inflammation was high across all three studies, with a clear homogeneity (I^2^: 0%) from all studies. Studies also had good precision and validity, as evidenced by their documented protocols. In addition, a very low significant mean difference (0.09) was reported across the studies on this study outcome, as measured by the DAS, increasing the strength of the quality of the outcome.

**TABLE 4:** GRADE evidence profile: pain, physical function, quality of life, health-related quality of life, disability, stiffness, and inflammation.

Quality assessment						Summary of findings number of patients					
Study outcome [no. of studies]	Limitation	Inconsistency	Indirectness	Imprecision	Publication bias	Control	Intervention	RR/SMD (95% CI)	Heterogeneity (I^2^)	Difference (95% CI)	Quality
Pain, PF/Q/H- 24 months ACR20 [4]	No serious	No serious	No serious	No serious	Significant bias	241/2020	133/2020	RR 1.86	77%	Significant	†††B
	limitations	inconsistency	indirectness	imprecision				(1.20e2.98)			Moderate
Pain, PF/Q/H at 24-month ACR50 [4]	No serious	No serious	No serious	No serious	Significant bias	75/2020	156/2020	RR 2.46	74%	Significant	†††B
	limitations	inconsistency	indirectness	imprecision				(1.30e4.66)			Moderate
Pain, PF/Q/H at											
24 months ACR70 [4]	No serious	No serious	No serious	No serious	Undetected	36/2020	88/2020	RR 2.63	47%	Not significant	††††
	limitations	inconsistency	indirectness	imprecision				(1.35e5.12)			High
Disability at											
at 24 weeks HAQDI [3]	No serious	No serious	No serious	No serious	Undetected	-----	-----	SMD -0.08	58%	Not significant	††††
	limitations	inconsistency	indirectness	imprecision				(-0.25e0.10)			High
Stiffness 24wkDAS [3]	No serious	No serious	No serious	No serious	Undetected	-----	-----	SMD 0.09	0%	Not significant	††††
	limitations	inconsistency	indirectness	imprecision				(-0.01e0.19)			High

Abbreviations: GRADE, Grading of Recommendations Assessment; Development, and Evaluation; CI, confidence interval; RR, risk ratio; SMD, standardized mean difference; PF/Q/H, physical function, quality of life, and health-related quality of life, as measured by the American College of Rheumatology (ACR) criteria; †, one rating present; B, one rating absent; [ ], contains the number of studies.

Overall, the quality of evidence of study outcomes ranged closely from moderate to high quality across the studies and with minimal detection of study bias, but with substantial study homogeneity. The prevailing high quality of evidence lends credence to the quality of assessment of the outcomes included in this review of high-quality RCTs and subsequent meta-analysis.

## Discussion

In recent years, a growing body of research has focused on therapeutic exercises and pharmacological interventions for treating RA. This systematic review evaluated the efficacy of these interventions compared to untreated groups or placebos, providing insights into their impact on managing RA. Although relatively sparse data are available for studies on therapeutic exercise interventions, several publications on pharmacological interventions for RA exist. However, exercise interventions seem to be receiving attention recently, perhaps due to increased awareness and researchers’ interest in this area (Teuwen et al., 2024; Da Silva et al., 2013; Lourenzi et al., 2017). This was revealed by several recent articles that emanate from different exercise interventions (Teuwen et al., 2024; Da Silva et al., 2013; Lourenzi et al., 2017). Previous publications were dominated by pharmacological interventions (Klareskog et al., 2004; Kremer et al., 2006; Schiff et al., 2014; Emery et al., 2008; Genovese et al., 2008; Smolen et al., 2008; Fleischmann et al., 2009; Gabay et al., 2013; van Vollenhoven et al., 2009; Smolen et al., 2009; Nishimoto et al., 2004). Most pharmacological interventions involved the treatment of RA with abatacept (Kremer et al., 2006), tocilizumab (Genovese et al., 2008), infliximab, sulfasalazine, hydroxychloroquine (van Vollenhoven et al., 2009), certolizumab pegol (Fleischmann et al., 2009), adalimumab (Gabay et al., 2013), etanercept, and methotrexate alone or in combination with the other pharmacological interventions (Emery et al., 2008). In contrast, therapeutic exercise programs largely comprised endurance exercise (Teuwen et al., 2024), sensorimotor training (Da Silva et al., 2013), and resistance exercise (Lourenzi et al., 2017). However, more research articles are expected on exercise interventions to strengthen the efficacy pool of this method. Furthermore, patients with functional disability due to RA have been shown to respond positively to exercise therapy (Teuwen et al., 2024). Emphatically, in clinical guidelines on the management of RA, exercise therapy is recommended in addition to pharmacological treatment (Gartlehner et al., 2006), and in some reviews (Daien et al., 2017; Roodenrijs et al., 2021), a position largely supported by evidence in this review (Teuwen et al., 2024; Rodríguez Sánchez-Laulhé et al., 2022; Da Silva et al., 2013; Häkkinen et al., 2004; Lamb et al., 2015; Van Den Berg et al., 2006; Lourenzi et al., 2017). This review observes a significant improvement in patient-specific complaint activity, ranked 1 (PSC1) for the intervention group, compared to the placebo group (Teuwen et al., 2024). Studies have shown that pharmacological treatment strategies for RA have improved drastically over the last decades (Emery et al., 2008). Currently, fewer patients with RA have unsatisfactory control of disease activity, as illustrated by 5–20% of patients with RA, thereby fulfilling the criteria for difficult-to-treat (D2T) RA in clinical studies (Roodenrijs et al., 2021).

To further define the evidence of the efficacies of pharmacological and exercise interventions of the included articles using a cumulative effect size, a meta-analysis led to a larger sample size and pooled effects of statistical significance. Four pharmacological studies (Genovese et al., 2008; Fleischmann et al., 2009; Gabay et al., 2013; van Vollenhoven et al., 2009) that investigated the improvement in clinical outcomes using the ACR tool for adult patients with RA were included in the meta-analysis. The improvement in clinical outcomes was observed in most pharmacological interventions, as recorded in the order of ACR 20, ACR 50, and ACR 70. These clinical improvement hierarchies show ACR 70 > ACR 50 > ACR 20 improvement levels. However, the percentage of participants with clinical improvements is always relatively higher in ACR 20 than in ACR 50, and there is a greater reduction in ACR 70 as a smaller population of participants achieves a high ACR level.

Moreover, a cumulative effect size of 1.89 was found for four reported pharmacological interventions (Genovese et al., 2008; Fleischmann et al., 2009; Gabay et al., 2013; van Vollenhoven et al., 2009) for ACR 20. As expected, there was incremental improvement in effect size, going further to ACR 50 and ACR 70 with 2.46 and 2.63, respectively. This effect size represents an improvement in the overall clinical outcomes for the treatment groups over the control groups. Furthermore, it was observed that certolizumab pegol (Fleischmann et al., 2009) shows the highest contribution (3.50) to the overall effect size of the clinical outcome performance, followed by tocilizumab in combination with DMARD (2.44) (Genovese et al., 2008), which, in turn, was followed by tocilizumab monotherapy (1.33) (Gabay et al., 2013), with the least being infliximab (1.31) (van Vollenhoven et al., 2009) for the period of 24-month interventions, as revealed by ACR 20. A similar trend was observed in ACR 50 and ACR 70 for this period. This review also documented that combination therapy demonstrated greater efficacy than monotherapy for most interventions.

### Pharmacological mechanisms of interventions and classification

The mechanisms of action (MOA) of pharmacological interventions often relate to their potential differences in clinical efficacy. This is detailed as follows, with emphasis on interventions found in this review.

### Biologic DMARD mechanisms of action

Biologic DMARDs (bDMARDs) target specific molecules (cytokines or cell surface receptors) central to the inflammatory and autoimmune cascade of RA. Their primary mechanisms can be clearly separated into three categories as follows.

1. TNF-α inhibitors: Their MOA is to neutralize tumor necrosis factor alpha (TNF-α), a major pro-inflammatory cytokine. This reduces systemic inflammation, decreases joint damage, and inhibits the destructive effects of joint-resident cells such as fibroblasts. The key examples are adalimumab, etanercept, and infliximab (Smolen et al., 2020).
2. IL-6 receptor inhibitors: Their MOA involves blocking the interleukin-6 (IL-6) receptor, preventing IL-6—a critical driver of inflammation, systemic symptoms (such as fever and fatigue), and acute-phase reactants (e.g., CRP), from binding and signaling. The key examples include tocilizumab and sarilumab (Aletaha and Smolen, 2018; Kawczak et al., 2025).
3. T-cell modulators: They interfere with the required co-stimulation signal needed for full T-cell activation. In particular, they block the CD80/CD86 pathway from binding to the CD28 receptor on T-cells, which dampens the entire adaptive immune response cascade that drives RA pathogenesis. Abatacept is a key example (Kawczak et al., 2025).

### Relationship between mechanism of action and efficacy (ACR response)

Differences in MOA theoretically influence efficacy, particularly for patients who have not responded adequately to initial TNF-inhibitor therapy.

1. TNF inhibitors: Generally, they are considered the first-line bDMARD due to extensive evidence and high overall efficacy in RCTs. High ACR response rates (ACR50/ACR70) are achievable, especially in biologic-naïve patients (Smolen et al., 2020).
2. IL-6 inhibitors: These agents have demonstrated high efficacy, often comparable to or slightly exceeding that of TNF inhibitors in some studies, particularly in patients with high levels of systemic inflammation (elevated C-reactive protein, CRP). They have also shown competitive ACR20/ACR50/ACR70 rates as monotherapy, where TNF inhibitors often require a combination with methotrexate (Aletaha and Smolen, 2018). The distinct MOA makes them a highly effective option for TNF-inhibitor non-responders (Aletaha and Smolen, 2018; Kawczak et al., 2025).
3. T-cell modulators (abatacept): By targeting T-cell activation, this class intervenes earlier in the immune cascade. Abatacept has demonstrated consistent efficacy, although its ACR response rates may be marginally lower than those of TNF inhibitors and IL-6 inhibitors in head-to-head comparisons in biologic-naive patients. However, its distinct MOA makes it a valuable and reliable option for patients who have failed prior therapy from either TNF or IL-6 classes (Saki et al., 2021).

### Efficacy interpretation of pharmacological interventions

The importance of the pharmacological and clinical significance of incremental ACR improvement cannot be overemphasized. This is, therefore, discussed as follows.

Interpreting ACR response increments (ACR20/50/70): The ACR response criteria measure the percentage improvement in the number of tender and swollen joints, along with the improvement in at least three of five other disease activity measures. The incremental jump from ACR20 to ACR70 following pharmacological interventions, as observed in the studies, has profound significance, discussed as follows.

Pharmacological significance: ACR20 represents a minimal clinical response, often achievable by modest suppression of TNF- and IL-6. It validates that the drug engages its target and is biologically active but does not signify deep remission or effective disease control. ACR70 represents a major clinical response (good improvement). This level of efficacy indicates near-complete suppression of the inflammatory cascade driving RA. Achieving ACR70 requires potent pharmacological action that not only blocks initial pro-inflammatory signals but also sustains that suppression to allow significant clinical and functional recovery (Carpenter et al., 2016). This level is pharmacologically challenging, often requiring agents with a superior affinity, stability, and delivery profile.

Clinical significance and treatment decision-making: Clinically, ACR20 is insufficient. A patient achieving only ACR20 would likely still experience significant pain, fatigue, and radiographic progression (joint damage). Treatment would typically be intensified or changed immediately (Smolen et al., 2020). Consequently, ACR70 and higher represents the ultimate therapeutic goal, closely correlates with clinical remission or low disease activity, and most importantly, signifies prevention of irreversible joint damage (radiographic non-progression) and long-term physical function preservation (Carpenter et al., 2016). Real-world decision-making is heavily influenced by the following: an ACR70 response justifies continuing a therapy, while a failure to progress beyond ACR20 after 3–6 months warrants a switch to an agent with a different pharmacological mechanism (e.g., from a TNF inhibitor to an IL-6 inhibitor or T-cell modulator).

### Pharmacological rationale for combination versus monotherapy

The decision to use combination therapy, typically a bDMARD plus MTX, versus monotherapy is a cornerstone of RA treatment and is driven by pharmacological synergy. There are essential differences in both pharmacological rationale and clinical relevance of combination compared to monotherapy, described as follows.

Monotherapy (e.g., IL-6i alone): The pharmacological rationale for monotherapy is the non-immunogenic target/mechanism. Certain bDMARDs, including the IL-6 receptor inhibitors (e.g., tocilizumab) and the soluble TNF receptor fusion protein (etanercept), tend to have lower inherent immunogenicity or their MOA is less reliant on combination therapy for optimal efficacy. Furthermore, IL-6 receptor inhibitors, due to their potent and distinct MOA, have demonstrated equivalent efficacy as monotherapy compared to combination therapy in some studies, offering an important option for patients who cannot tolerate MTX (Brodszky et al., 2017).

Combination therapy (e.g., TNF-i + MTX): The rationales for the use of combination therapy in RA management are as follows. First, combination therapy aids synergistic efficacy. MTX, a conventional synthetic DMARD (csDMARD), works primarily by inhibiting purine synthesis, leading to anti-inflammatory and immunosuppressive effects. When combined with a bDMARD (which targets specific cytokines), the combination achieves multilevel blockade of the inflammatory and proliferative pathways (Jyssum et al., 2024; Bre et al., 2006). The clinical relevance of this is that it leads to higher ACR response rates (ACR50/70) and superior radiographic non-progression compared to either drug alone in most trials (Bre et al., 2006). This is the standard of care for most patients initiating bDMARD therapy. Second, combination therapy causes reduced immunogenicity. MTX often helps decrease the formation of anti-drug antibodies (ADAs) against the bDMARD (particularly monoclonal antibodies such as adalimumab or infliximab). ADAs can reduce the effective concentration of the biologic agent, leading to a loss of response. MTX’s immunosuppressive effect protects the bDMARD, thereby maintaining pharmacological potency and sustained efficacy (Jyssum et al., 2024). Thus, combination therapy is preferred for biologics with known immunogenic potential to ensure long-term drug survival and effectiveness.

### Safety considerations with pharmacological interventions for RA

The safety and tolerability of pharmacological interventions for RA, especially biologics, are clinically relevant. There is a need to emphasize major adverse risks associated with bDMARDs while focusing on the underlying pharmacological reasons, as follows.

1. Risk of serious infection (SI): The primary safety concern with all bDMARDs is an increased risk of serious bacterial, fungal, and opportunistic infections, most notably tuberculosis (TB) reactivation. Pharmacological rationale for this is admissible to the fact that these drugs function by disrupting the immune response. For TNF inhibitors, TNF is crucial for the formation and maintenance of granulomas, the cellular wall-offs the immune system uses to contain pathogens such as *Mycobacterium tuberculosis.* By neutralizing TNF, these drugs compromise granuloma integrity, leading to the reactivation of latent TB (Dixon et al., 2007). For IL-6 inhibitors, IL-6 plays a role in inflammatory signaling that triggers symptoms including fever, which can mask the early signs of infection. Blocking IL-6 can result in less apparent symptoms of infection (e.g., lower fever or less pronounced leukocytosis), leading to delayed diagnosis and potentially higher severity of the infection (Sepriano et al., 2023).
2. Infusion reactions: These are common, although often non-serious, adverse events specific to the administration route of bDMARDs. Infusion reactions (IV administration, e.g., infliximab) are acute events occurring during or shortly after infusion. The pharmacological rationale is frequently immunological, often caused by the formation of ADAs against the fully humanized or chimeric protein. These immune complexes trigger mast cell and basophil degranulation, leading to symptoms such as flushing, dyspnea, or anaphylaxis (Jyssum et al., 2024). This mechanism explains why pre-medication (antihistamines and corticosteroids) is often used. For injection site reactions (subcutaneous administration, e.g., adalimumab), these are local reactions (erythema, pain, and swelling) at the injection site. The pharmacological rationale is often non-immunological and may be related to the local effect of the drug formulation (e.g., concentration, pH, or preservative agents including citrate) rather than a systemic allergic response (Jyssum et al., 2024).
3. Immunological adverse events: These involve the immune system but are distinct from infections. Cytopenia may occur since agents such as IL-6 inhibitors (tocilizumab) are associated with neutropenia and thrombocytopenia. The pharmacological rationale follows that since IL-6 is known to regulate hematopoiesis, its blockade alters the bone marrow environment and platelet production, leading to measurable decreases in neutrophil and platelet counts in some patients (Sepriano et al., 2023). Furthermore, demyelinating events/psoriasiform reactions may occur. Although this is rare, TNF inhibitors are associated with the risk of new-onset or exacerbation of demyelinating disorders and psoriasiform skin lesions. The exact mechanism is debated, but it is hypothesized that the suppression of TNF disrupts the balance of the immune regulatory network, potentially favoring the development of auto-antibodies or an inflammatory state driven by other cytokines (such as Type 1 interferons) that affect the skin or central nervous system (Dang et al., 2023).

Pharmacological sources of heterogeneity: The observed variation in ACR20/50/70 response rates is often rooted in the drug’s properties and how it interacts with the disease. The pharmacological factors with the effect on efficacy and heterogeneity are explored as follows.

1. Drug class/mechanism: Different classes (e.g., TNF vs. IL-6 vs. T-cell modulators) would perform differently in patient subsets based on their dominant inflammatory pathways. For instance, a patient driven by a high IL-6 signature may show a poor response to a TNF inhibitor but a robust ACR70 response to an IL-6 blocker (Aletaha and Smolen, 2018). This variation is a feature of targeted therapy; the choice of target (TNF, IL-6, or CD80/86) dictates the likelihood of response in a heterogeneous patient population (Aletaha and Smolen, 2018).
2. Immunogenicity: For monoclonal antibodies (e.g., adalimumab, and infliximab), the formation of ADAs leads to sub-therapeutic drug concentrations over time, causing a secondary loss of efficacy and greater outcome variance in real-world studies (Jyssum et al., 2024). Hence, studies linking low drug trough levels (due to ADAs or rapid clearance) to lower ACR responses demonstrate a pharmacological basis for heterogeneity in long-term outcomes.
3. Dose: Subtle differences in dosing schedules can influence sustained drug levels (pharmacokinetics). Inadequate trough concentrations lead to greater variability in clinical response across centers and patient groups. Therapeutic drug monitoring studies highlight that patient metabolism and compliance create a wide range of actual drug exposures, which, in turn, causes the reported heterogeneity in ACR outcomes (Smolen et al., 2020).

Patient-level and disease stage variation: The efficacy of a drug is highly dependent on the baseline state of the patient’s disease, which contributes to trial heterogeneity.

1. Disease duration and stage: Patients with early RA (within the disease duration year) typically show a higher and faster ACR response to bDMARDs than those with long-standing, erosive RA (Aletaha and Smolen, 2018). This is because joint damage and the establishment of non-reversible pathogenic mechanisms in late-stage disease limit the full potential of anti-inflammatory drugs.
2. Prior treatment history: Biologic-naive patients generally show superior ACR response rates compared to those who have failed one or more prior biologics (known as switching patients). Based on history, reporting this outcome clarifies the variance: a lower ACR50 rate in a study of switching patients is an expected outcome, not a sign of poor drug efficacy across all populations (Smolen et al., 2020).

Publication bias and contextualization: The variance reported in the literature is partially due to publication bias, where pharmaceutical-sponsored trials often select highly homogenous, treatment-naive populations (efficacy) for initial reporting, leading to higher ACR rates. Conversely, registry or observational studies reflect real-world effectiveness in heterogeneous, multi-drug-failure populations, resulting in lower and more variable ACR outcomes (Smolen et al., 2020).

### Pharmacological mechanisms of exercise synergy

Therapeutic exercise does not only improve muscle strength but also acts as a non-pharmacological anti-inflammatory agent that complements the action of DMARDs through several biological pathways. These are explained as follows.

1. Modification of inflammatory pathways (the anti-inflammatory effect): This can be achieved in one of the following ways.
  1.1 Myokines and anti-inflammatory signaling: During and after exercise, skeletal muscles release small proteins called myokines (e.g., IL-6 and IL-15). Although IL-6 is generally pro-inflammatory when chronically elevated in RA, the transient, exercise-induced surge of IL-6 is followed by the stimulation of potent anti-inflammatory cytokines, notably interleukin-1 receptor antagonist (IL-1ra) and IL-10 (Pedersen, 2013). By increasing systemic IL-1ra and IL-10, exercise works in parallel with DMARDs (which block TNF or IL-6 signaling) to create a broader, multi-target anti-inflammatory environment. This dual suppression may help achieve and maintain deeper states of remission (ACR70/DAS28-remission) than drug therapy alone (Osthoff et al., 2018; Metsios and Kitas, 2018).
  1.2 Downregulation of pro-inflammatory cytokines: Regular exercise can reduce the systemic levels of major RA-driving cytokines, including TNF and CRP (Hu et al., 2021). This reduction reduces the overall inflammatory burden, potentially increasing the effective concentration of the pharmacological agent (DMARD) available to target remaining local inflammation in the synovium, rather than being “soaked up” by widespread systemic inflammation (Osthoff et al., 2018; Metsios and Kitas, 2018).
2. Improvement of muscle metabolism and insulin sensitivity:

Rheumatoid arthritis is associated with chronic inflammation that causes peripheral insulin resistance and leads to muscle atrophy (cachexia) and metabolic syndrome, often worsened by corticosteroid use. Resistance and aerobic exercise improve insulin sensitivity in muscle cells (Bilberg et al., 2024). Improved muscle metabolism enhances overall physical function and combats the extra-articular (non-joint) manifestations of RA, reinforcing the clinical gains achieved by the pharmacological suppression of joint inflammation (Osthoff et al., 2018; Metsios and Kitas, 2018).

### Limited Sub-Saharan Africa (SSA) RCTs and pharmacological relevance

The scarcity of RCTs from Sub-Saharan Africa (SSA) is a significant limitation that creates a knowledge gap concerning the pharmacological profile of DMARDs in this region. This is because pharmacological responses, including drug metabolism and immune-mediated adverse events, can be influenced by genetic polymorphisms. Findings from predominantly Caucasian populations (where most RA trials are conducted) may not perfectly translate to the genetically diverse populations in SSA (Adelowo et al., 2021). The efficacy, dosing, and safety of DMARDs, especially synthetic DMARDs (sDMARDs) including methotrexate, which are metabolized by specific enzymes, require local validation. Moreover, patients in SSA may present with different RA phenotypes, often characterized by later presentation, more severe disease, and higher rates of comorbidities, including endemic infections (ORGANISERS, 2013). These factors profoundly alter the risk–benefit ratio and the pharmacological interaction of immunosuppressive biologics, requiring unique safety monitoring strategies not captured by Western trials.

### Constraints on translation: accessibility and affordability

The primary constraint on translating high-efficacy trial findings to real-world pharmacological practice in LMICs is the lack of access to and affordability of biologics. The high cost makes bDMARDs practically unavailable for the vast majority of patients. This forces clinical practice to rely almost entirely on csDMARDs, even in cases of severe or refractory disease where biologics are the evidence-based standard of care (Sepriano et al., 2023; Adelowo et al., 2021). Another constraint is the issue of limited safety monitoring. The use of potent immunosuppressants including biologics necessitates robust safety infrastructure (e.g., screening for latent tuberculosis and frequent lab monitoring for cytopenias). Where health systems lack these resources, the pharmacological risks (adverse events) often outweigh the potential benefits, making the drug unsuitable for use even if it were available (Adelowo et al., 2021).

Real-world pharmacological practice in SSA: In practical terms, the pharmacological landscape in SSA is dictated by resource-constrained effectiveness rather than maximal efficacy. Moreover, delayed access is a challenge in these areas such that when biologics are used, they are typically reserved as a last-resort intervention for the most severe, erosive cases, rather than being used early in the disease course, as advocated by the window of opportunity concept proven in RCTs (ORGANISERS, 2013). This delay undermines the potential for long-term radiographic and functional preservation.

### Clinical translation and pharmacological guidance

1. Translating efficacy to clinical practice: The clinical interpretation of efficacy results must be framed in the context of the “Treat-to-Target” (T2T) strategy, which is the guiding principle of modern RA management. T2T demands rapid and aggressive therapeutic changes until a state of low disease activity (LDA) or remission is achieved, which are the true clinical endpoints. This is further buttressed as follows. First, the target in clinical management is not ACR20, but remission (ACR70). Hence, clinically, an ACR70 response (or achieving a DAS28 score) is the critical goal because it correlates strongly with radiographic non-progression (preventing irreversible joint damage) and optimal long-term functional status. The primary pharmacological purpose of any first-line therapy is to achieve this deep response as quickly as possible (Smolen et al., 2020; Sepriano et al., 2023). Second, clinical management is affected by the window of opportunity, in which case, clinical practice requires that the most robust pharmacological response is obtained when therapy is initiated early in the disease course (months). This translational finding suggests that treatment decisions are urgency-driven, aiming to use the most effective drug combination upfront to halt the disease before permanent structural damage occurs (Aletaha and Smolen, 2018). Third, a crucial translational step is incorporating safety data. For instance, the high risk of serious infection (including TB reactivation) associated with TNF inhibitors necessitates mandatory pre-screening in clinical practice before the drug can be safely initiated (Dixon et al., 2007). Decision-making on the choice of pharmacological intervention, therefore, requires consideration of these multi-pronged factors.
2. Pharmacological considerations guiding first- versus second-line therapy: The sequential choice of therapy is guided by the pharmacological principles of efficacy maximization and mechanism diversification in the event of treatment failure.

First-line choice: This entails maximizing initial efficacy and safety. The decision for initial systemic therapy is primarily guided by historical evidence of safety, efficacy, and cost. The initial pharmacological backbone remains MTX, often combined with other csDMARDs (e.g., sulfasalazine/hydroxychloroquine). This is based on its well-established, multilevel immunosuppressive mechanism and favorable risk-to-cost ratio (Smolen et al., 2020). If the patient has poor prognostic factors or fails MTX, the first biologic is typically a TNF inhibitor. This preference is guided by the vast extent of long-term efficacy and safety data from numerous RCTs and registries, making it the most validated pharmacological starting point for biologics.

Second-line choice: Here, the focus is on mechanism diversification. If a patient shows an inadequate clinical response (i.e., fails to achieve remission) to the first-line biologic after 3–6 months, the next therapeutic choice is driven by a change in the drug’s MOA. This is the core pharmacological principle of second-line treatment: First, if the patient fails a TNF inhibitor, the pharmacological conclusion is that the TNF pathway is not the dominant driver of their inflammatory disease or that ADAs have rendered the drug ineffective (Jyssum et al., 2024). Subsequently, the clinician must then switch to a drug with a fundamentally different MOA to target an alternative pathogenic pathway. Pharmacological results guide this switch: switch to the IL-6 inhibitor (tocilizumab) targets a distinct pathway (IL-6), offering a non-cross-reactive mechanism. Clinical trial data have shown high efficacy for IL-6 inhibitors even in patients who have failed TNF drugs (Aletaha and Smolen, 2018). Furthermore, switch to the T-cell modulator (abatacept) targets an earlier stage of the immune response (co-stimulation), offering another distinct MOA that has proven effective in the TNF failure population (Kawczak et al., 2025). In essence, clinical translation entails using the observed ACR failure (e.g., no ACR50 after 3 months) as a pharmacological biomarker, indicating the need to target a new, non-redundant immune pathway for successful disease control.

### Therapeutic exercise in rheumatoid arthritis

Therapeutic exercise encompasses a structured, planned, and supervised program of physical activity specifically designed to improve functional capacity, muscle strength, joint mobility, and cardiorespiratory fitness in patients with RA (Teuwen et al., 2024; Rodríguez Sánchez-Laulhé et al., 2022; Da Silva et al., 2013; Häkkinen et al., 2004; Lamb et al., 2015; Cerasola et al., 2023). This approach moves beyond general physical activity; it is a prescribed, evidence-based treatment that acts as a non-pharmacological anti-inflammatory agent and is synergistic with DMARD therapy. It is safe, even in patients with active disease, when implemented and monitored correctly.

### Components of therapeutic exercise and specific effects in rheumatoid arthritis

Therapeutic exercise protocols for RA are typically divided into three primary categories based on their mechanisms and effects:

1. Resistance exercise (strength training): This component involves activities designed to increase muscle strength, power, and mass by working muscle groups against an opposing force (e.g., weights and bands). The primary goal is to counteract rheumatoid cachexia—the loss of lean muscle mass common in RA—and improve muscle function (Baillet et al., 2012; Lourenzi et al., 2017; Bilberg et al., 2024). It enhances functional and clinically significant improvements in isometric and isokinetic strength and reduces self-reported disability (Cairns and McVeigh, 2009; Boniface et al., 2020). Moreover, resistance exercise fosters metabolic counteraction as it improves muscle function and stability, directly mitigating the debilitating effects of chronic inflammation and muscle atrophy (Osthoff et al., 2018).
2. Aerobic exercise (endurance training): This involves sustained, rhythmic activities that enhance cardiorespiratory fitness (e.g., walking, cycling, and swimming). This is vital for managing chronic fatigue and reducing the increased cardiovascular risk associated with RA (Ye et al., 2022; Boniface et al., 2020). Aerobic exercise induces the transient release of myokines (cytokines secreted from skeletal muscle), notably IL-6 (Smolen et al., 2008; Metsios and Kitas, 2018). This exercise-induced IL-6 surge acts in an anti-inflammatory manner by stimulating the production of anti-inflammatory cytokines, such as IL-1ra and IL-10, thereby working in parallel with DMARDs to suppress systemic inflammation (Benatti and Pedersen, 2015). Furthermore, this exercise reduces systemic levels of pro-inflammatory markers such as TNF (Roodenrijs et al., 2021; Smolen et al., 2020) and CRP over time, contributing to overall disease control (Benatti and Pedersen, 2015).
3. Range-of-motion (ROM) exercises (flexibility training): These are gentle movements and stretching designed to maintain or increase the flexibility of joints and surrounding soft tissues (Metsios and Kitas, 2018). They directly target joint stiffness, a hallmark symptom of active RA, especially morning stiffness. In addition, they aid mobility preservation by preventing joint contractures and maintaining maximum possible joint movement, which is essential for preserving long-term functional independence, despite joint damage (Hu et al., 2021; Cerasola et al., 2023).

### Translational implication: optimizing pharmacological and exercise synergy

The clinical translation of therapeutic exercise lies in its ability to synergistically enhance the effects of pharmacological treatment, primarily by modifying the inflammatory and metabolic environment. The optimal exercise prescription is individualized, disease-activity informed, and balanced with the ongoing pharmacological regimen.

1. Optimal exercise prescription: The choice of exercise type and intensity in RA is directly informed by the patient’s current Disease Activity Score (DAS28), making it a dynamic prescription. During high disease activity/flare, the goal is to prevent contractures and disuse atrophy without mechanically exacerbating active synovitis, and the focus is primarily on ROM exercises and low-impact aquatic aerobics. Intensity should be very low (Ye et al., 2022). In low disease activity/remission, however, the goal of therapeutic exercise is to maximize muscle mass, increase cardiorespiratory fitness, and trigger systemic anti-inflammatory myokine release, which supports the drug-induced remission. At remission, the focus is on combined high-intensity resistance and aerobic training. Resistance should be 60–80% of one-repetition maximum (Lemmey et al., 2009).
2. Synergy with pharmacological Intervention: The combination of therapy is optimal when exercise and drugs target different, but complimentary, pathological mechanisms. Pharmacological intervention creates a state of low systemic inflammation that allows the patient to safely tolerate and benefit from the mechanical loading required for effective resistance and aerobic exercise. The mechanism involves direct target blockade of specific inflammatory pathways to halt synovial inflammation and joint erosion (Smolen et al., 2020). Therapeutic exercise, in synergy, enhances overall physical function (e.g., HAQ-DI) beyond what drugs can achieve alone. It is safe and effective only when the pharmacological agent has already suppressed the underlying severe inflammation. Metabolic and endocrine modulation (e.g., myokines and insulin sensitivity) can help counteract rheumatoid cachexia, cardiovascular risk, and fatigue (Benatti and Pedersen, 2015). In translational practice, the success of the pharmacological regimen (remission) is the prerequisite that unlocks the maximal benefits of a therapeutic exercise program, leading to superior long-term function and quality of life, in line with World Health Organization’s sustainable development goal 3 of good health and wellbeing (Long, 2022).

### Study limitations

This review is not without its limitations. A notable gap exists in the geographic distribution of the included studies, with limited or no representation from LMICs, particularly those in SSA. Additionally, there is a lack of comparative studies evaluating pharmacological interventions alongside exercise therapy, despite existing evidence supporting the effectiveness of both approaches. Considerable variability was observed in sample sizes and outcome measures, with different scales used across studies. In contrast to the relatively abundant literature on pharmacotherapy, the limited data available on therapeutic exercise hinder a balanced comparison of their effectiveness. Moreover, the absence of SSA RCTs significantly limits the findings’ generalizability to those settings. This review did not assess potential adverse effects or safety concerns associated with the interventions in the studies reviewed, but the safety aspects and concerns of interventions were emphasized in the discussion. Despite these limitations, the review offers a valuable foundation for understanding the effectiveness of pharmacological and exercise-based interventions in treating RA.

## Conclusion

The review of the available data reveals that pharmacological or exercise interventions (as single therapies) have improved outcomes in patients with RA. The effective combinations of pharmacotherapies, such as certolizumab pegol and methotrexate, along with etanercept and methotrexate, have shown more promising effects than monotherapy. This is very crucial as methotrexate or DMARD has always been utilized as the baseline drug for RA management; therefore, its combination with other pharmacotherapies can easily be harnessed. The evidence of improved quality of life and functional ability observed in different exercise therapies in this review suggests that combining exercise programs with pharmacotherapy could yield even more efficacious results than their separate interventions on patients with RA. The observed dearth of clinical trials of Sub-Saharan African origin, along with the paucity of documented studies comparing pharmacological and therapeutic exercise interventions along common outcomes, highlights gaps that may benefit from future research by experts in the field.

## Data Availability

The original contributions presented in the study are included in the article/Supplementary Material; further inquiries can be directed to the corresponding author.

## Supplementary material

SUPPLEMENTARY DATA SHEET 1 PRISMA Checklist.

SUPPLEMENTARY TABLE 1 Data extraction sheet.

SUPPLEMENTARY TABLE 2 Quality appraisal tool.

SUPPLEMENTARY TABLE 3 Kappa details of authors’ agreement.

SUPPLEMENTARY FILE 1 Search strategy.
