# Supplementary material for: Efficacy of pharmacological interventions and therapeutic exercises for rheumatoid arthritis: a systematic review and meta-analysis
Source: Front Pharmacol. 2025 Nov 20;16:1686128. doi: 10.3389/fphar.2025.1686128 (PMC12676119; doi:10.3389/fphar.2025.1686128)
Supplement: Supplementary file 3 [file Supplementaryfile1.pdf]

SEARCH STRATEGY: WOS, CINAHL and SCOPUS

Total: 1881

Duplicate: 248

Web of Science (WOS)

S/N	KEYWORDS	SEARCH STRATEGY	RESULTS
#1	Rheumatoid arthritis	TS=("Rheumatoid arthritis" OR "inflammatory arthritis") and Preprint Citation Index (Exclude – Database)	322,911
#2	Rheumatoid arthritis	(TS=(Rheumatism OR Rheumatics OR Rheuma OR Rheumatical)) AND TS=(arthritis) and Preprint Citation Index (Exclude – Database)	63,876
#3	#1 OR #2	#1 OR #2 and Preprint Citation Index (Exclude – Database)	341,751
#4	Pharmacological intervention	TS=(Pharmacological intervention OR Drug intervention OR Biological DMARD* OR targeted synthetic DMARD* OR Janus Kinase Inhibitor* OR JAK inhibitor* OR Glucocorticoid* OR Corticosteroid* OR Cox-2 inhibitor OR Steroids OR NSAID* OR Aspirin OR Ibuprofen OR Diclofenac OR Ketorolac OR Celecoxib OR Methotrexate OR Leflunomide OR Hydroxychloroquine OR Sulfasalazine OR TNF inhibitor OR TNFi* OR antitumor necrosis factor OR Interleukin-6 receptor antagonist OR Interleukin-6 receptor blocker OR IL-6R inhibitor OR IL-6 antagonist OR tocilizumab OR Interleukin-1 receptor antagonist OR IL-1R antagonists OR T-Cell Costimulation Modulator OR T cell costimulatory signal protein OR Cytotoxic T-Lymphocytes associated proteins OR Abatacept OR Upadacitinib OR Baricitinib OR Filgotinib OR Tofacitinib OR CD20 Inhibitor OR B-cell depleting agents OR CD20-directed cytolytic antibodies OR Cytotoxic T-lymphocyte-associated antigen 4 OR CTLA4-Ig) and Preprint Citation Index (Exclude – Database)	2,156,297
#5	Exercise therapy	TS=("exercise therapy*" OR Aerobiosis OR exercise OR "Aerobic exercise" OR "Plyometric Exercise") and Preprint Citation Index (Exclude – Database)	1,083,125
#6	#3 AND #4 AND #5	#3 AND #4 AND #5 and Preprint Citation Index (Exclude – Database)	919
#7	FILTERS	#3 AND #4 AND #5 and Preprint Citation Index (Exclude – Database) and 2024 or 2022 or 2023 or 2021 or 2020 or 2019 or 2018 or 2017 or 2016 or 2015 or 2014 or 2013 or 2012 or 2011 or 2010 or 2009 or 2008 or 2007 or 2006 or 2005 or 2004 (Publication Years)	702

CINAHL

S/N	KEYWORDS	SEARCH STRATEGY	RESULTS
#1	Rheumatoid arthritis	"Rheumatoid arthritis" OR “inflammatory arthritis”	(34,100)
#2	Rheumatoid arthritis	((Rheumatism OR Rheumatics OR Rheuma OR Rheumatical)) AND (arthritis)	(23,591)
#3	#1 OR #2	#1 OR #2	(22,442)
#4	Pharmacological intervention	Pharmacological intervention OR Drug intervention OR Biological DMARD* OR targeted synthetic DMARD* OR Janus Kinase Inhibitor* OR JAK inhibitor* OR Glucocorticoid* OR Corticosteroid* OR Cox-2 inhibitor OR Steroids OR NSAID* OR Aspirin OR Ibuprofen OR Diclofenac OR Ketorolac OR Celecoxib OR Methotrexate OR Leflunomide OR Hydroxychloroquine OR Sulfasalazine OR TNF inhibitor OR TNFi* OR antitumor necrosis factor OR Interleukin-6 receptor antagonist OR Interleukin-6 receptor blocker OR IL-6R inhibitor OR IL-6 antagonist OR tocilizumab OR Interleukin-1 receptor antagonist OR IL-1R antagonists OR T-Cell Costimulation Modulator OR T cell costimulatory signal protein OR Cytotoxic T-Lymphocytes associated proteins OR Abatacept OR Upadacitinib OR Baricitinib OR Filgotinib OR Tofacitinib OR CD20 Inhibitor OR B-cell depleting agents OR CD20-directed cytolytic antibodies OR Cytotoxic T-lymphocyte-associated antigen 4 OR CTLA4-Ig	(131,254)
#5	Exercise therapy	"exercise therapy*" OR Aerobiosis OR exercise OR "Aerobic exercise" OR "Plyometric Exercise"	(216,724)
#6	#3 AND #4 AND #5	#3 AND #4 AND #5	(55)
#7	FILTERS	S3 AND S4 AND S5<br>Limiters - Publication Date: 20040101-20241231	(43)

S/N	KEYWORDS	SEARCH STRATEGY	RESULTS
#1	Rheumatoid arthritis	TITLE-ABS-KEY ("Rheumatoid arthritis" OR "inflammatory arthritis")	237,677 results
#2	Rheumatoid arthritis	(TITLE-ABS KEY ((rheumatism OR rheumatics OR rheuma OR rheumatical)) AND TITLE-ABS-KEY ((arthritis)))	38,861 results
#3	#1 OR #2	(TITLE-ABS-KEY ("Rheumatoid arthritis" OR "inflammatory arthritis")) AND ((TITLE-ABS-KEY ((rheumatism OR rheumatics OR rheuma OR rheumatical)) AND TITLE-ABS-KEY ((arthritis))))	30,413 results
#4	Pharmacological intervention	TITLE-ABS-KEY (pharmacological AND intervention OR drug AND intervention OR biological AND dmard* OR targeted AND synthetic AND dmard* OR janus AND kinase AND inhibitor* OR jak AND inhibitor* OR glucocorticoid* OR corticosteroid* OR cox-2 AND inhibitor OR steroids OR nsaid* OR aspirin OR ibuprofen OR diclofenac OR ketorolac OR celecoxib OR methotrexate OR leflunomide)	19 results
#5	Exercise therapy	TITLE-ABS-KEY ("exercise therapy*" OR aerobiosis OR exercise OR "Aerobic exercise" OR "Plyometric Exercise")	846,562 results
#6	#3 AND #4 AND #5	((TITLE-ABS-KEY ("Rheumatoid arthritis" OR "inflammatory arthritis")) AND ((TITLE-ABS-KEY ((rheumatism OR rheumatics OR rheuma OR rheumatical)) AND TITLE-ABS-KEY ((arthritis))))) AND (TITLE-ABS-KEY (pharmacological AND intervention OR drug AND intervention OR biological AND dmard* OR targeted AND synthetic AND dmard* OR janus AND kinase AND inhibitor* OR jak AND inhibitor* OR glucocorticoid* OR corticosteroid* OR cox-2 AND inhibitor OR steroids OR nsaid* OR aspirin OR ibuprofen OR diclofenac OR ketorolac OR celecoxib OR methotrexate OR leflunomide)) AND (TITLE-ABS-KEY ("exercise therapy*" OR aerobiosis OR exercise OR "Aerobic exercise" OR "Plyometric Exercise"))	1 results
#7	FILTERS	((TITLE-ABS-KEY ("Rheumatoid arthritis" OR "inflammatory arthritis")) AND ((TITLE-ABS-KEY ((rheumatism OR rheumatics OR rheuma OR rheumatical)) AND TITLE-ABS-KEY ((arthritis))))) AND (TITLE-ABS-KEY (pharmacological AND intervention OR drug AND intervention OR biological AND dmard* OR targeted AND synthetic AND dmard* OR janus AND kinase AND inhibitor* OR jak AND inhibitor* OR glucocorticoid* OR corticosteroid* OR cox-2 AND inhibitor OR steroids OR nsaid* OR aspirin OR ibuprofen OR diclofenac OR ketorolac OR celecoxib OR methotrexate OR leflunomide)) AND	1 results

		(TITLE-ABS-KEY ("exercise therapy*" OR aerobiosis OR exercise OR "Aerobic exercise" OR "Plyometric Exercise")) AND PUBYEAR = 2023	

SCOPUS
